# Challenges and Solutions in pgRNA Measurement: Toward Improved Monitoring of Hepatitis B Therapy

**DOI:** 10.3390/pathogens15020153

**Published:** 2026-01-31

**Authors:** Zhenkun Zhu, Jin Wu, Jinyuan Li, Tao Wu

**Affiliations:** 1 Department of Clinical Laboratory Medicine, Third Clinical Medical College of Ningxia Medical University, People’s Hospital of Ningxia Hui Autonomous Region, No. 255 Zhengyuan North Street, Jinfeng District, Yinchuan 750002, China; zhuzhenkun@hbuas.edu.cn (Z.Z.); 250312020736@nxmu.edu.cn (J.W.); 250312020737@nxmu.edu.cn (J.L.); 2 Xiangyang Central Hospital, Affiliated Hospital of Hubei University of Arts and Science, Xiangyang 441021, China

**Keywords:** hepatitis B virus, pregenomic RNA, cccDNA, sample preservation, detection methodologies

## Abstract

Hepatitis B virus (HBV) pregenomic RNA (pgRNA), transcribed directly from nuclear covalently closed circular DNA (cccDNA), is an essential component in viral replication. The synthesis and encapsidation of pgRNA depend significantly on the transcriptional activity of cccDNA, making serum pgRNA a recently recognized non-invasive biomarker for evaluating cccDNA activity. However, its clinical application is limited by factors including preanalytical variables, methodological inconsistencies in detection, and a lack of standardization in quantification. This review provides an overview of the biological origins of pgRNA and its critical role in the HBV replication cycle, highlighting the stability challenges encountered during the collection, processing, and storage of plasma/serum samples. Furthermore, it analyzes recent significant advancements in pgRNA detection technologies, encompassing modified reverse transcription quantitative polymerase chain reaction (RT-qPCR), nucleocapsid-captured methodologies, automated testing platforms, multiplex digital PCR, isothermal amplification, and clustered regularly interspaced short palindromic repeats-based assays. A comparison of these technologies revealed that discrepancies in pgRNA quantification arise primarily from variations in sample processing and measurement systems, rather than from inherent biological limitations. Therefore, establishing standardized sample handling procedures, harmonized detection methods, and unified measurement systems is imperative before pgRNA can be reliably applied to monitor treatment, guide cessation decisions, or evaluate cure in chronic hepatitis B.

## 1. Introduction

Hepatitis B virus (HBV) infection remains a major global public health concern, with particularly high prevalence rates observed in Africa and Asia. Recent epidemiological data indicate that approximately 4.1% of the global population is infected with HBV, resulting in over 300 million affected individuals [1]. Chronic HBV infection significantly increases the risk of liver fibrosis, cirrhosis, and hepatocellular carcinoma (HCC) and remains a leading cause of HCC-related mortality globally [2]. Despite considerable advancements in antiviral therapies aimed at reducing viral replication and improving clinical outcomes, attaining a functional cure for chronic hepatitis B (CHB) remains a significant challenge.

**Figure 1:**
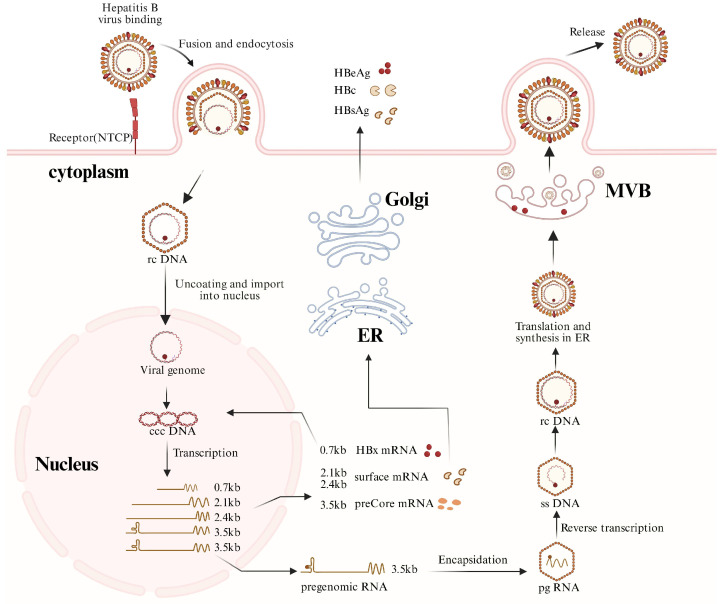
HBV replication cycle.

HBV is classified within the “Hepadnaviridae” family, characterized by mature virions that possess a partially double-stranded relaxed circular DNA (rcDNA) of approximately 3.2 kilobases in length (Figure 1). Upon viral entry, mediated by the liver-specific receptor sodium taurocholate co-transporting polypeptide, the rcDNA is transported into the nucleus, where it undergoes repair to form covalently closed circular DNA (cccDNA). This cccDNA persists as minichromosome-like structures and serves as the primary template for viral transcription. Host RNA polymerase (Pol) II transcribes this template, yielding five species of overlapping 3′-terminal viral transcripts, comprising the 3.5 kb precore mRNA (pcRNA) and pregenomic RNA (pgRNA), the 2.4/2.1 kb surface protein mRNAs, and the 0.7 kb HBx mRNA. Among these transcripts, the pgRNA is specifically encapsulated into nucleocapsids and functions as the template for reverse transcription, hence playing a crucial role in the HBV replication cycle. In the cytoplasmic nucleocapsids, HBV Pol catalyzes the reverse transcription of the encapsulated pgRNA to synthesize new rcDNA. This newly synthesized rcDNA is subsequently enveloped and released through the multivesicular body pathway, leading to the development of mature viral particles. Infected hepatocytes release significant amounts of subviral particles lacking nucleic acids and nucleocapsids, and genome-deficient empty virions and unenveloped nucleocapsids, all of which contribute to the complex composition of circulating viral components [3,4,5]. 

This figure illustrates the major steps of the HBV life cycle, including viral entry via the sodium taurocholate co-transporting polypeptide (NTCP), de-envelopment, conversion of nuclear rcDNA into cccDNA, transcription of viral RNAs, protein translation, encapsidation of pregenomic RNA (pgRNA), reverse transcription, and assembly and secretion of viral particles. pgRNA transcribed from nuclear cccDNA is selectively packaged into nucleocapsids and serves as the template for reverse transcription. In addition to mature virions, subviral particles, empty virions, and RNA-containing particles may also be released. (Abbreviations: NTCP, sodium taurocholate co-transporting polypeptide; rcDNA, relaxed circular DNA; cccDNA, covalently closed circular DNA; pgRNA, pregenomic RNA; ssDNA, single-stranded DNA; dslDNA, double-stranded linear DNA; ER, endo plasmic reticulum; MVB, multivesicular body.). Created with BioRender.com (https://biorender.com, accessed on 30 December 2025).

In chronic HBV infection, intranuclear cccDNA is widely acknowledged as the primary molecular determinant that facilitates viral replication and post-treatment recurrences. However, existing antiviral therapies exhibit minimal efficacy in directly eradicating cccDNA, and its long-term stability presents a significant challenge to achieving a functional cure [6]. Intrahepatic cccDNA levels are regarded as crucial prognostic indicators for evaluating the response to antiviral treatment [7]; their direct quantification in clinical settings has been challenging due to the heterogeneous distribution within liver tissue and the requirement for invasive liver biopsies for detection [8,9]. As a result, there is an urgent need for the development of non-invasive alternative biomarkers that precisely indicate cccDNA transcriptional activity and are suitable for peripheral blood testing, which has emerged as a focal point in contemporary HBV research and clinical practice.

Therefore, HBV pgRNA has garnered significant academic interest owing to its unique biological characteristics. pgRNA is the direct transcriptional product of intranuclear cccDNA and fulfills dual roles: it encodes the HBV Pol and acts as a template for reverse transcription. The generation, encapsulation, and reverse transcription are intricately dependent on the transcriptional activity of cccDNA. Clinically, serum HBV DNA levels below the detection threshold often indicate effective suppression of the reverse transcription process; however, this does not necessarily imply the elimination of cccDNA. Residual cccDNA can sustain low-level transcription and may lead to virological rebound following the cessation of nucleos(t)ide analog therapy [10,11]. Consequently, exclusive dependence on HBV DNA monitoring is inadequate for a comprehensive evaluation of disease prognosis and therapeutic risks.

Recent studies indicate that serum HBV pgRNA exhibits significant clinical potential in three primary areas: first, as an indirect marker of the transcriptional activity of intrahepatic cccDNA; second, in predicting the risk of virological relapse after the cessation of nucleos(t)ide analogs (NAs); third, in facilitating the assessment of seroconversion or the loss of HBeAg or HBsAg [12]. However, the absence of a standardized consensus regarding detection procedures, management of preanalytical variables, quantitative standardization, and a clinical interpretation framework for pgRNA remains a barrier to its widespread clinical adoption. This article aims to systematically review the molecular origin and biological properties of HBV pgRNA, with an emphasis on its stability in plasma/serum samples and recent advancements in detection methodologies. This will provide a comprehensive evaluation of the potential utility of pgRNA in the clinical management of chronic hepatitis B (CHB) and in curative treatment strategies, aiming to furnish a systematic reference for the transition of pgRNA from an investigational marker to a clinically applicable biomarker.

## 2. Molecular Biological Functions of HBV pgRNA

The replication of the HBV is characterized by protein-primed reverse transcription, in which the pgRNA performs dual roles: it acts as a translation template for synthesizing viral core proteins and Pol and as a template for reverse transcription activities [13,14]. Initially, the cytoplasmic pgRNA facilitates the translation of core protein and Pol. The Pol binds to the ε (epsilon) stem–loop structure at the 5′ end of the pgRNA, forming the pgRNA-Pol complex, which is subsequently packaged into the nucleocapsid through recruitment by core proteins, therefore initiating the reverse transcription process [15,16,17,18]. In the preliminary stage of reverse transcription, the terminal protein (TP) domain of Pol acts as a protein primer, synthesizing a nascent negative-strand DNA of 3–4 nucleotides on the bulged sequence of the ε structure [19]. This short DNA strand is subsequently translocated to the direct repeat 1 (DR1) region at the 3′ end of the pgRNA, where it hybridizes with the first three nucleotides and facilitates the synthesis of the negative-strand DNA. During the elongation of the negative-strand DNA, the RNase H activity of Pol progressively degrades the pgRNA template, retaining only a short RNA fragment that contains the 5′ DR1 region [20,21]. Following its synthesis, this RNA fragment is translocated to the DR2 region of the negative-strand DNA, where it serves as a primer for the synthesis of positive-strand DNA, ultimately leading to the formation of rcDNA. Occasionally, the RNA primer fails to effectively transfer the template to DR2, leading to the production of double-stranded linear DNA (dslDNA) as a replication by-product. On rare occasions, the RNA primer does not successfully complete the template transfer to DR2, leading to the production of double-stranded linear DNA (dslDNA) as a replication by-product [22,23].

## 3. Impact of Preanalytical Variables on Plasma/Serum pgRNA Stability and Test Results

The precision of HBV pgRNA detection in plasma/serum is significantly influenced by the detection platform and the preanalytical variables that precede sample testing. These variables encompass the entire process from post-collection storage to processing and pre-detection phases, including aspects such as preservation temperature, duration under ex vivo conditions, sample processing approaches, and the intrinsic degradation characteristics of pgRNA. Considering that pgRNA sustains a dynamic transcription-degradation equilibrium in vivo, its stability ex vivo is not entirely constant. Variations in preanalytical procedures among various studies may result in discrepancies in test results. The cumulative effect of these preanalytical parameters is essential in ascertaining the stability and reproducibility of plasma/serum pgRNA quantification outcomes.

### 3.1. Storage Conditions and Stability of pgRNA in Plasma/Serum Specimens

For an extended period, serum HBV DNA and hepatitis B surface antigen (HBsAg) levels have been extensively employed to evaluate viral replication status and the efficacy of antiviral therapy [24]. During NAs therapy, HBV DNA replication is often significantly inhibited, frequently falling below the lower limit of detection (LoD). Meanwhile, cccDNA within hepatocyte nuclei can maintain low-level transcriptional activity, making conventional serum markers insufficient for accurately representing the actual viral activity status. The concept of serum HBV RNA/pgRNA as a biomarker was originally proposed based on longitudinal clinical observations in nucleos(t)ide analog-treated patients. In a seminal study, van Bömmel et al. demonstrated that serum HBV RNA remains detectable despite profound suppression of serum HBV DNA, and that its dynamics are associated with treatment-induced HBeAg seroconversion, suggesting that circulating pgRNA reflects ongoing cccDNA transcription rather than residual viral replication [25,26]. It should be noted that these early studies were hypothesis-generating in nature: while they established the biological plausibility of serum pgRNA as a transcriptional marker, they did not address assay comparability, transcript heterogeneity, or standardization across platforms—limitations that underlie much of the subsequent variability reported in the literature. In this context, clarifying the stability of pgRNA in clinical specimens and its sensitivity to preanalytical variables has become an essential issue in its clinical translation process.

RNA molecules are inherently susceptible to deterioration during quantitative detection methods, with their stability influenced by various factors, including temperature, storage duration, freeze–thaw cycles, ribonuclease (RNase) contamination, and the presence of PCR inhibitors [27]. Before the routine clinical application of nucleic acid testing for RNA viruses, including HIV and HCV, the stability of their plasma RNA was systematically assessed. Previous studies have demonstrated that plasma HIV RNA remains stable for approximately three days when stored at room temperature (25 °C) or at 4 °C [28]. Conversely, HBV RNA maintains stability for at least four days at 4 °C, although its stability significantly diminishes when stored at 23 °C or 37 °C [29]. These findings offer essential insights for enhancing preanalytical conditions for RNA biomarkers.

Conversely, although HBV pgRNA has garnered increasing attention due to its capacity to indicate the transcriptional activity of intrahepatic cccDNA, its stability in plasma or serum specimens has not been comprehensively evaluated. Unlike most RNA viruses, circulating pgRNA predominantly exists in a form encapsulated within nucleocapsids, a molecular characteristic that may enhance its stability in peripheral blood to some extent. The present understanding of pgRNA composition is inconsistent, potentially including various subtypes such as the 3.5 kb full-length pgRNA, 3′-truncated transcripts, and splice variants (spliced pgRNA) [30,31]. Importantly, HBV RNA splicing and the existence of structurally distinct transcripts are not newly recognized phenomena. Pioneering molecular studies conducted in the late 1980s and 1990s first characterized the generation and structural features of spliced HBV RNAs, demonstrating that these transcripts are produced during viral replication and can accumulate in infected hepatocytes [32,33]. Subsequent work further suggested that truncated or structurally heterogeneous HBV RNA species may coexist with full-length pgRNA. From a methodological perspective, such transcript heterogeneity implies that quantitative detection strategies targeting different genomic regions may differentially capture these RNA species, thereby introducing systematic variability that is not solely attributable to RNA degradation or storage-related instability. The stability variations among these subtypes under storage and processing conditions remain inadequately clarified. This uncertainty exacerbates the potential influence of preanalytical variables on pgRNA quantification results.

Valerie et al. conducted a systematic evaluation of the impact of preanalytical variables on the stability of pgRNA in plasma samples from 26 patients infected with HBV, employing the Roche cobas^®^ 6800/8800 investigational HBV RNA assay [34]. Nevertheless, it should be emphasized that these stability observations are intrinsically conditional on the specific analytical workflow, assay design, and operational definitions applied. Differences in sample matrix, baseline pgRNA concentration, target-region selection, and the operational definition of storage duration or freeze–thaw cycles may contribute to divergent conclusions across studies. Indeed, reports describing substantial pgRNA stability under controlled conditions do not necessarily contradict studies reporting marked signal loss following repeated freeze–thaw procedures, but rather highlight the strong context dependence of stability assessments. The findings demonstrated that samples with initial concentrations exceeding 100 copies/mL remained consistently detectable after 48 h of storage within a temperature range of 4–42 °C. Among the various temperature conditions evaluated, storage at 4 °C yielded the most stable quantitative results, exhibiting the least variability. Therefore, from the perspective of clinical detection protocols, it is recommended to prioritize storage at 4 °C for short-term preservation of samples.

In further assessments of long-term stability, this study found that HBV RNA remains stable for at least 30 days when stored at −20 and 4 °C. Even under conditions of 25 or 37 °C, pgRNA remains detectable for at least 7 days. Moreover, after undergoing a minimum of three freeze–thaw cycles, pgRNA levels in samples did not exhibit a significant decline. These findings suggest that HBV pgRNA demonstrates relatively high stability across diverse storage conditions when collected, processed, and preserved according to standardized protocols, thereby offering essential methodological support for its detection in clinical settings [34].

Collectively, available evidence indicates that HBV pgRNA in plasma/serum can exhibit a higher degree of stability than initially expected under well-defined and standardized pre-analytical conditions; however, variability in storage protocols, assay design, and transcript composition remains a major source of inter-study inconsistency (Table 1). Taken together, comparative analyses across studies employing different sample handling protocols and measurement platforms indicate that a substantial proportion of the reported variability in pgRNA quantification is attributable to methodological heterogeneity rather than intrinsic biological instability of pgRNA itself [28,29,34]

We note that the literature is not fully consistent regarding freeze–thaw effects. While some studies (including automated workflows under defined protocols) reported limited quantitative change after several freeze–thaw cycles, Ohlendorf et al. observed a 1–2 log decline in plasma/serum after repeated freeze–thaw. This discrepancy is likely driven by methodological and operational differences rather than a single biological explanation, including assay design and target region, sample matrix and processing (serum vs. plasma), baseline RNA concentration (low-copy samples being more vulnerable to stochastic loss), and non-uniform definitions of a ‘freeze–thaw cycle’ (e.g., thaw duration, mixing, and time at ambient temperature). Therefore, pgRNA stability should be viewed as protocol-dependent rather than universally robust

Collectively, the body of existing evidence indicates that HBV pgRNA is a relatively stable RNA biomarker, demonstrating robust detection stability when preanalytical variables are adequately controlled. Ensuring cross-study comparability of pgRNA quantitative data necessitates the standardization of detection technologies and target regions and hinges critically on the implementation of uniform protocols for sample collection, processing, and storage conditions. Therefore, systematic management of preanalytical variables should be considered a fundamental component, equally as crucial as detection methodologies, in the clinical translation of pgRNA.

### 3.2. Sample Processing Methods for pgRNA and Their Impact on Test Results

Given that pgRNA exhibits relatively satisfactory stability across various storage conditions, a crucial factor influencing test outcomes is the sample processing procedure, particularly focusing on RNA extraction techniques and strategies to mitigate DNA interference. The accuracy of HBV pgRNA test results is highly dependent on the sample preprocessing protocols, as these methods significantly impact RNA recovery efficiency, the extent of DNA interference, and the stability of detection specificity. Consequently, sample processing techniques constitute an essential component that must be carefully addressed in methodological research and the clinical application of pgRNA detection.

The conventional RNA extraction primarily utilizes the phenol-chloroform method, which is theoretically capable of yielding relatively intact total RNA. This technique involves labor-intensive procedures, is highly sensitive to experimental conditions, often results in substantial RNA loss in samples with low RNA content, such as plasma or serum, and exhibits significant inter-assay variability. Existing studies demonstrate that, under identical initial sample volume conditions, the recovery rate of pgRNA extracted using the phenol-chloroform method is approximately 62%, significantly lower than the 85% recovery rate attained with the magnetic bead-based method (*p* < 0.01) [35]. Moreover, residual organic solvents from the phenol-chloroform extraction method may inhibit subsequent reverse transcription or amplification activities, thereby compromising the accuracy of quantitative results.

Recently, RNA extraction methods employing silica or magnetic bead carriers have increasingly become the standard. The magnetic bead-based approach enhances RNA enrichment by facilitating the reversible binding of nucleic acids to solid-phase carriers, thus enhancing recovery efficiency and significantly reducing the risk of RNase contamination. Additionally, this method facilitates the standardization and automation of procedures, making it highly appropriate for high-throughput processing of clinical samples. Thus, in both methodological research and the clinical detection of pgRNA, the magnetic bead-based method is widely recognized as superior to conventional organic extraction techniques.

In addition to conventional RNA extraction techniques, recent research has investigated detection strategies that bypass the need for RNA extraction. One such approach involves a nucleic acid hybridization-based method for detecting pgRNA, which enables direct capture and identification through the construction of oligonucleotide probes that target conserved pgRNA regions and facilitate a sandwich hybridization reaction on a solid surface. This approach has demonstrated high detection performance in validation studies, achieving sensitivity and specificity rates of 91.47% and 90.63%, respectively [36]. Although these approaches theoretically reduce the loss and variability associated with RNA extraction processes, further evaluation is required to evaluate their resistance to interference in complex serum matrices and their quantitative stability.

The removal of DNA is a critical step in sample processing to ensure the specificity of pgRNA detection. The substantial sequence overlaps between pgRNA and HBV DNA indicate that any residual DNA can significantly disrupt the quantitative assessment of pgRNA. Research has demonstrated that DNase I treatment can effectively eliminate the interference caused by HBV DNA, thereby enhancing the specificity of pgRNA detection from approximately 75% to 95% (*p* < 0.01) [37]. However, whereas DNase treatment enhances specificity, it may also increase the risk of RNA degradation and introduce additional operational variability. To reduce reliance on DNase treatment, some studies suggest selective detection of pgRNA through the design of amplification target regions. For instance, selective reverse transcription-polymerase chain reaction (RT-PCR) methods that focus on the poly(A) tail of pgRNA using specific primers facilitate the specific amplification of pgRNA without requiring DNase I treatment, achieving a detection limit of approximately 100 copies/mL [37]. This approach delegates the control of specificity to the amplification stage, thereby simplifying experimental procedures and minimizing the potential for interference from residual DNA.

The methods employed for processing samples containing pgRNA significantly affect test results. It is essential to thoroughly evaluate extraction techniques, DNA removal strategies, and their combinations with detecting platforms and specific application settings. These factors must be explicitly defined and regulated in methodological research.

### 3.3. Mechanisms of pgRNA Degradation

Beyond the technical variability introduced during sample processing, pgRNA naturally exists in a dynamic balance between transcription and degradation within the host, with its stability being actively regulated by both host and viral stimuli. The stability of pgRNA in plasma or serum is influenced by storage conditions and handling procedures and intricately regulated by host factors and viral proteins during its degradation. The degradation mechanism of pgRNA demonstrates the complex interplay between host antiviral defenses and viral replication methods, which has significant implications for understanding its stability in vivo and the variability observed in detection results.

At the host level, interferon-induced ribonucleases are essential in the degradation of pgRNA. The interferon-stimulated gene 20 (ISG20) functions as a 3′–5′ exoribonuclease that specifically identifies and binds to the ε (epsilon) structural element of pgRNA through its C-terminal ExoIII domain, thus promoting the selective degradation of pgRNA. Functional analyses indicate that ISG20 knockdown results in an approximate twofold increase in pgRNA levels [38], highlighting its essential role in constraining the accumulation of HBV transcripts. Furthermore, the host signaling molecule MyD88 has been implicated in the inhibition of HBV replication by promoting pgRNA degradation. In vitro experiments have demonstrated that overexpression of MyD88 significantly reduces the half-life of pgRNA from approximately 24 to 6 h [39]. These findings indicate that innate immune signaling pathways exert antiviral effects through inflammatory responses and contribute to the control of viral replication by directly modulating viral RNA stability.

At the viral level, the RNase H activity of the HBV Pol is essential in the degradation of pgRNA. RNase H facilitates pgRNA template strand degradation during reverse transcription, and any dysfunction in its activity may cause abnormal accumulation of pgRNA within nucleocapsids or viral particles, resulting in an approximate threefold increase in pgRNA levels [40]. This observation indicates that pgRNA degradation is not solely a consequence of host defense mechanisms but is a fundamental aspect of the reverse transcription process within the HBV life cycle. Moreover, specific host factors have been identified that target pgRNA at distinct sites to facilitate its degradation. NgAgo specifically targets the P-2166 site on pgRNA, facilitating its degradation and leading to a decrease in pgRNA levels by approximately 1.5 log_10_ copies/mL following treatment [41]. These findings indicate that the stability of pgRNA is regulated by complex, multi-level molecular mechanisms, and its degradation is characterized by particular sequence and structural specificity.

The degradation of pgRNA is influenced by the interaction between host immune regulation and viral replication mechanisms. This intrinsic dynamic stability impacts the in vivo levels of pgRNA and underpins the variability in test outcomes across various disease states and sample conditions.

## 4. HBV pgRNA Detection Technology: Platform Evolution, Standardization, and Clinical Translation

A wide range of pgRNA assays have been reported, but their reported analytical performance (e.g., LoD) is difficult to compare directly because studies differ in sample matrices, extraction volumes, target regions, DNA-removal steps, and calibration materials. For clinical translation, the more decisive question is therefore not which assay reaches the lowest LoD under its own conditions, but which strategy can deliver reproducible longitudinal monitoring with inter-laboratory comparability. In the following sections, we discuss detection platforms primarily through a clinical-translation lens—standardization/traceability, robustness to pre-analytical variation, and feasibility of automation—while distinguishing approaches that are mainly research-enabling from those that are plausible candidates for multicenter clinical studies.

To align with the stated goal of clinical translation, we apply a clinical-translation filter to distinguish platforms that are plausible candidates for multicenter studies from those that are primarily research-enabling or accessibility-oriented. Specifically, the key criteria include: (i) pre-analytical robustness and defined handling requirements; (ii) analytical specificity (RNA–DNA discrimination and target-region clarity); (iii) standardization/traceability and inter-laboratory comparability (availability of calibrators or traceable reference systems); (iv) automation/closed workflow feasibility for routine operation; and (v) evidence base in longitudinal clinical cohorts or multicenter settings. Methods lacking traceability or standardized workflows are discussed as valuable research tools, but not positioned as immediate candidates for regulatory-grade clinical implementation

### 4.1. Detection Strategies for Specificity and Biological Identity

In clinical settings, HBV pgRNA, which predominantly constitutes serum/plasma HBV RNA, is utilized as an indicator of cccDNA transcriptional activity and residual replication status. Significantly, even when NA therapy reduces HBV DNA levels beyond the lower limit of detection, pgRNA may remain detectable, providing stratification and predictive significance. Various pgRNA detection platforms exhibit considerable differences regarding target region selection, DNA interference control, automation level, and clinical applicability. Table 2 presents the primary technical characteristics and application scenarios.

We acknowledge a key limitation in the current literature: there is no widely adopted head-to-head comparison of multiple pgRNA platforms using the same well-characterized clinical sample panel processed under identical pre-analytical conditions. Consequently, sensitivity claims and LoD values summarized across studies should be interpreted as within-study performance rather than as a basis for strict cross-platform ranking. To enable meaningful comparisons, future multicenter evaluations should standardize at minimum the specimen type (serum/plasma), handling time/temperature, extraction input volume, target region definition, DNA-interference control, and a shared calibration material (or traceable reference system).

### 4.2. Target Region Design-Based One-Step Quantitative Detection

The quantitative detection of HBV pgRNA is primarily performed using quantitative RT-qPCR. However, the significant sequence overlaps between pgRNA and rcDNA—where pgRNA is approximately 1.1 times the full length of the HBV genome—poses issues for conventional amplification target sequences in distinguishing RNA from DNA at the molecular level. This constitutes the fundamental methodological challenge for achieving pgRNA-specific detection [42].

To reduce DNA interference, contemporary RT-qPCR techniques typically incorporate an additional DNase I digestion step before reverse transcription to eliminate HBV DNA from extracted nucleic acid mixtures [43]. However, in practical applications, DNase treatment often fails to achieve complete DNA removal, with residual rcDNA potentially leading to false-positive results or quantitative overestimation. Moreover, this supplementary DNA removal step inevitably results in RNA loss, reduces detection sensitivity, and increases the complexity of experimental procedures, time requirements, and inter-assay variability [25]. Accordingly, the advancement of methods for the specific amplification of pgRNA independent of DNase treatment has become a crucial focus for optimizing RT-qPCR systems.

Recent studies have presented a streamlined one-step qRT-PCR method that enables the selective amplification of pgRNA by targeting its distinctive poly(A)/polA tail sequence, thus eliminating the need for DNA digestion before reverse transcription [37]. This methodological innovation simplifies the workflow and may reduce RNA loss associated with DNase treatment; however, its effective specificity in clinical specimens remains dependent on target-region design, transcript composition in serum/plasma, and explicit controls that rule out co-amplification of other HBV RNAs. It is essential to acknowledge that the quantitative outcomes of such one-step assays are partially contingent upon the ability of the selected target region to encompass various pgRNA structural forms, including truncated transcripts or splice variants. Therefore, the methodological benefits and potential biases are closely associated with the optimization of the target region and the challenges of transcript heterogeneity, which are discussed elaborately in subsequent sections.

Importantly, ‘DNase-free’ does not equate to ‘pgRNA-exclusive’. Poly(A)-targeting designs can, in principle, capture other polyadenylated HBV transcripts (e.g., preC mRNA and related RNAs), and the magnitude of this contribution may vary by HBeAg status and transcript composition in patient samples. In addition, quantification can be biased by transcript integrity: assays anchored near the 3′ end may under-represent 3′-truncated or fragmented pgRNA, whereas assays anchored elsewhere may shift the balance between sensitivity and isoform coverage. Therefore, claims of DNase-independent specificity should be supported by minimum controls, including (i) explicit assessment of DNA carryover (e.g., spike-in or DNA-only controls), (ii) strategies to estimate or constrain preC mRNA contribution (e.g., differential/dual-target designs), and (iii) reporting of the target region relative to known truncation/splice patterns.

### 4.3. Nucleocapsid Capture-Based Detection of Encapsidated RNA

Beyond the differentiation of RNA from DNA, a crucial methodological challenge in HBV pgRNA detection involves ascertaining whether the detected RNA is directly associated with the viral replication process. Given that only pgRNA encapsulated within nucleocapsids undergoes subsequent reverse transcription, distinguishing between encapsidated and non-encapsidated RNA during the detection stage is critically important. This distinction is essential for clarifying the mechanisms of HBV replication and assessing the efficacy of antiviral drugs.

Preliminary investigations employed techniques such as the RNase protection assay and an in-gel combined Northern blot to differentiate between encapsulated and unencapsulated RNA. However, these methods were constrained by complex workflows, limited throughput, and dependence on radioactive probes, which obstructed their ability to fulfill the requirements for high-throughput or dynamic quantitative analysis. To address these challenges, Ryu et al. [44] developed the nucleocapsid-captured quantitative RT-qPCR (NCC RT-qPCR) method. This novel technique utilizes anti-HBc antibody-coated 96-well plates to specifically capture viral nucleocapsids. Subsequent lysis facilitated by proteinase K, together with DNase I treatment to eliminate replicative intermediate DNA, enables the quantitative detection of encapsulated pgRNA using one-step SYBR RT-qPCR. Methodological evaluations demonstrated that the results obtained from NCC RT-qPCR were highly consistent with those from conventional in-gel analysis, thereby affirming its specificity and reliability in the quantification of encapsidated RNA.

Compared to radioactive detection techniques, NCC RT-qPCR offers significant advantages by eliminating the need for membrane transfer and isotope handling, thus enabling the simultaneous detection of multiple samples within microplate systems. This innovation significantly enhances experimental throughput and operational safety, establishing it as an essential instrument for the examination of HBV encapsidation processes and the high-throughput evaluation of antiviral drugs. The current application of this method is predominantly limited to in vitro HBV replication models. Further systematic evaluation is necessary to determine its relevance to clinical specimens, including plasma or serum, and its reproducibility across different laboratories and potential for standardization.

### 4.4. Automation and Result Comparability in Detection Platforms

#### 4.4.1. Simultaneous Amplification and Testing (SAT) System

In employing HBV pgRNA as a marker for cccDNA transcriptional activity for clinical stratification and therapeutic efficacy assessment, conventional RT-qPCR methodologies encounter significant methodological challenges, including the repeatability of detection processes, contamination control, and reliable detection in low-copy number scenarios. To address these issues, Hu et al. [45] conducted a systematic evaluation of the Simultaneous Amplification and Testing (SAT) system’s performance in quantifying serum HBV RNA, offering a direct comparison with conventional RT-qPCR methodologies.

The SAT system seamlessly integrates RNA extraction, reverse transcription, and amplification within a completely automated closed framework, producing amplification products of readily degradable RNA. This design substantially reduces the risk of aerosol contamination during procedures and minimizes inter-assay variation caused by manual operations. An assessment of the methodology revealed that the system achieves a detection limit of 50 copies/mL, with a linear range extending from 10^2^ to 10^8^ copies/mL, comparable to that of RT-qPCR. A comparative analysis between the two methods revealed a high degree of consistency (R^2^ = 0.90, Bland–Altman difference ranging from −0.95 to 1.00 log_10_ copies/mL) and exhibited exceptional repeatability (coefficient of variation approximately 4%). Notably, in certain serum samples negative for HBV DNA, the SAT system successfully identified low levels of pgRNA, demonstrating its superior sensitivity in detecting residual viral transcriptional activity.

At the clinical application level, Liu et al. [46] utilized SAT to perform a quantitative analysis of serum pgRNA in a cohort of 291 treatment-naïve patients with CHB. This study systematically analyzed the dynamic alterations in pgRNA levels during various stages of the disease’s natural history. The findings demonstrated that patients who were positive for hepatitis B e-antigen (HBeAg) exhibited significantly elevated pgRNA levels compared to their HBeAg-negative counterparts, with values of 6.82 versus 2.40 log copies/mL, respectively. Additionally, a progressive decline in pgRNA levels was observed as the disease progressed from the immune-tolerant phase to the inactive chronic phase, followed by a re-elevation during the immune reactivation stage. Serum pgRNA exhibited significant correlations with HBV DNA (r = 0.79) and HBsAg (r = 0.55), with the strongest linear correlation observed with HBV DNA, especially among HBeAg-negative individuals. Further analysis revealed that a pgRNA threshold of 2.11 log copies/mL effectively distinguished inactive carriers from immune-reactive CHB patients within the HBeAg-negative population, yielding an area under the curve (AUC) of 0.833, with a sensitivity of 84.2% and specificity of 73.7%. This approach surpassed the conventional diagnostic techniques that integrate “HBV DNA + HBsAg.”

Methodologically, the SAT system primarily identifies pgRNA transcribed from cccDNA before reverse transcription, thus providing a more accurate representation of intrahepatic viral transcriptional activity compared to assays that target reverse-transcribed products or integration-derived fragments. The automated, closed workflow of SAT markedly improves the stability of low-copy pgRNA detection and minimizes RNA loss and DNA interference. Synthesizing existing evidence, SAT represents a practical step toward more standardized workflows and may facilitate broader evaluation of pgRNA in clinical laboratories [47]; however, its role in routine testing remains conditional on harmonized target-region definitions, traceable calibration, and cross-platform comparability. However, its widespread clinical implementation remains contingent upon further standardization of target region selection, metrological standardization, and cross-platform comparability.

#### 4.4.2. Fully Automated Standardized Platforms and Metrological Traceability

Although serum HBV RNA, primarily in the form of pgRNA, is widely recognized as a potential non-invasive biomarker reflecting the transcriptional activity of intranuclear cccDNA, its clinical application has been significantly hindered by the lack of result comparability among various laboratories and analytical platforms. Despite improvement in sensitivity, the primary methodological obstacles hindering the progression of pgRNA from an investigational marker to a conventional clinical test are the lack of traceability and the absence of standardized quantitative measures.

To address these challenges, Yu et al. [48] developed the first standardized, fully automated serum HBV RNA quantitative detection system using the Cobas^®^ 6800/8800 high-throughput platform. This system was systematically validated to evaluate its viability as a surrogate marker for cccDNA transcriptional activity. It utilizes a reverse transcription amplification process that is compatible with HBV DNA detection modules, employing specific primers and probes targeting the preC/C region, thereby facilitating fully automated procedures from nucleic acid extraction to quantitative analysis. This automation markedly reduces errors and contamination risks associated with manual handling. The approach can identify pgRNA transcribed from cccDNA and its derivative transcripts, offering a robust technical basis for assessing the persistent transcriptional status of the virus.

In a clinical cohort study of 316 patients with CHB, serum HBV RNA levels exhibited a strong correlation with intrahepatic cccDNA content (r = 0.78, *p* < 0.001). Consistent findings have been reported in other independent cohorts, in which serum HBV RNA/pgRNA levels showed significant correlations with intrahepatic cccDNA across different treatment settings, including long-term nucleos(t)ide analog therapy and pegylated interferon–based regimens [49,50,53]. Furthermore, significant correlations were observed with HBV DNA (r = 0.86) and HBeAg levels. Longitudinal follow-up data from patients receiving NA therapy indicated that, although HBV DNA levels rapidly decreased to below the detection threshold, the decrease in HBV RNA levels was significantly slower. Some patients remained HBV RNA positive even after HBV DNA became undetectable, indicating that HBV RNA is a more sensitive marker of the transcriptional activity of residual cccDNA. During post-treatment follow-up, patients with detectable HBV RNA demonstrated a significantly higher risk of relapse compared to those who were RNA-negative (76.9% versus 11.1%), highlighting the potential utility of HBV RNA as a predictive biomarker for relapse at the level of clinical outcomes.

The study’s most significant contribution is the first introduction of HBV RNA test results into the international unit (IU/mL) system, calibrated to WHO international standards. This advancement ensures metrological traceability and inter-laboratory consistency of test results. The standardization strategy creates a methodological basis for globally consistent HBV RNA detection, enabling pgRNA quantification to attain comparability and a clinical interpretation framework analogous to that of HBV DNA for the first time. Consequently, this fully automated, high-throughput workflow provides an important enabling platform for evaluating serum HBV RNA within a more standardized measurement framework. While it strengthens the technical basis for multicenter comparability, positioning pgRNA as a routine clinical parameter remains premature without broader cross-platform harmonization, traceable reference materials, and prospective studies linking test results to clinical decision thresholds.

#### 4.4.3. Highly Sensitive Automated Detection Platforms

As pgRNA is increasingly acknowledged as a potential surrogate marker for the transcriptional activity of intranuclear cccDNA, there is a growing demand for extremely sensitive detection methods equipped with automated features. Conventional manual RT-PCR techniques, characterized by multi-step procedures and the need for supplementary DNA removal processes, are especially susceptible to variations in experimental conditions, impeding their widespread implementation in routine clinical practice. In response to the limitations in detection capabilities at low viral load thresholds, Abbott has developed a second-generation pgRNA detection system (v2) utilizing its m2000 automated nucleic acid detection platform [48]. This development aims to significantly enhance the detection performance of pgRNA for monitoring treatment efficacy and assessing treatment cessation.

By maintaining the dual-target RT-qPCR framework, this system has significantly decreased the lower detection limit from approximately 150 copies/mL to 10–22 copies/mL. This improvement was accomplished by the optimization of nucleic acid extraction volume, enzyme reaction systems, and data processing algorithms, while maintaining a high degree of consistency with the first-generation detection method (R^2^ ≈ 0.99). The enhanced sensitivity significantly increases the system’s ability to identify low-titer samples, providing significant advantages in monitoring residual viral transcriptional activity during NA therapy and following treatment cessation, especially within clinical ranges that approach conventional detection limits.

However, from a methodological standardization perspective, this system remains indirectly calibrated against international HBV DNA standards and lacks specific quantitative reference materials for HBV RNA. Furthermore, there is a continued deficiency in systematic comparative studies utilizing absolute quantification techniques, such as digital PCR or other automated platforms. The quantitative comparability across various laboratories and the detection coverage for different pgRNA isoforms necessitate further validation. Thus, although the Abbott (version 2) platform has achieved significant advancements in automation and detection sensitivity, it should be viewed as a strong adjunct to existing pgRNA detection systems in the low-copy range, rather than as a definitive solution that completes a “standardization closed-loop.” Future efforts should focus on developing RNA-specific reference materials, verifying cross-platform consistency, and ensuring metrological traceability to determine its definitive function within routine clinical testing systems. Overall, different pgRNA detection methodologies offer unique advantages and limitations regarding sensitivity, automation level, structural coverage capability, and standardization potential.

### 4.5. High-Resolution Quantification and Transcript Structural Coverage

#### Multiplex Overlapping Digital PCR (ddOTs)

The structural heterogeneity of transcripts, along with detection sensitivity, poses a significant methodological challenge that affects the accuracy of HBV pgRNA quantification [51,52]. The pgRNA does not exist as a singular molecular entity but rather coexists in multiple structural variants, including 3.5 kb full-length transcripts, 3′-truncated transcripts, and various splice variants. In this context, quantification methodologies that rely on a single amplification target site are prone to systematic underestimation due to the absence of specific transcripts or structural variations.

Digital PCR (dPCR) is considered an optimal technological platform for accurate quantification of pgRNA owing to its ability to conduct absolute quantification without standard curves, its robust tolerance to PCR inhibitors, and its high sensitivity in detecting low-abundance nucleic acids. However, when employing a single-target design, dPCR cannot fundamentally address the quantification bias stemming from the structural heterogeneity of pgRNA. In response to this limitation, Tang et al. [54] proposed the multiplex overlapping digital PCR strategy, termed digital droplet Overlapped Targets (ddOTs). This innovative approach entails designing numerous mutually overlapping amplification target sites across different regions of pgRNA, thus achieving comprehensive fragment-level coverage of the transcript.

The primary benefit of ddOTs is their multi-target overlapping design, which effectively mitigates the risk of missed detection due to 3′ or 5′ truncations. This design integrates diverse structural subtypes into a cohesive pgRNA signal, facilitating comprehensive quantification. The methodological evaluation demonstrated that the system attained a detection sensitivity of 2–5 copies per reaction, with a linear range extending from 5 to 106 copies. In low-copy samples, its quantitative results surpassed those of single-target RT-qPCR and single-target dPCR regarding accuracy and repeatability. Moreover, the ddOTs test results exhibited high concordance with Northern blot analysis of isolated nucleocapsid RNA, hence further validating its reliability in accurately representing the total pgRNA quantity.

Overall, ddOTs introduces a high-resolution methodological framework for pgRNA measurement that prioritizes “structural coverage” over “single-point sensitivity.” This method is especially efficacious for examining pgRNA heterogeneity and its correlation with cccDNA transcriptional activity. This strategy is currently more applicable to research-grade or reference-grade applications due to its throughput and operational complexity. The comprehensive pgRNA quantification it offers is fundamentally valuable for optimizing target regions, standardizing detection methods, and enabling cross-platform result comparisons.

### 4.6. Advanced Technologies for Point-of-Care and Accessibility-Oriented Approaches

#### 4.6.1. Immunochromatographic Test Based on Catalytic Hairpin Assembly

Conventional RT-qPCR systems struggle to meet the demands for rapid response and on-site testing; thus, developing simplified pgRNA detection methods that do not require complex instrumentation presents significant potential for application in primary-level laboratories and resource-constrained environments. Recently, enzyme-free isothermal signal amplification strategies have garnered significant interest. Notably, catalytic hairpin assembly (CHA) enables cyclic signal amplification through the interaction of two complementary DNA hairpin structures, offering advantages such as enzyme-free operation, mild reaction conditions, and reduced costs [55,56].

Building upon this principle, Chen et al. [57] developed a novel dual-mode pgRNA detection system that combines CHA with an immunochromatographic platform. This system includes a fluorescent lateral flow immunochromatographic assay (CHA-LFIA) and a colloidal gold immunochromatographic assay (CHA-GICA). In this framework, pgRNA functions as a trigger sequence, facilitating the sequential unfolding of hairpin probes H1 and H2, which subsequently form a cyclically amplifiable H1–H2 double-stranded complex. The signal readout is achieved through the immunochromatographic platform. The CHA-LFIA method enables semi-quantitative detection using fluorescent microspheres after approximately 30 min of reaction at 37 °C, whereas the CHA-GICA method offers visual interpretation through colorimetric bands. The entire detection process is streamlined into a single tube, obviating the need for thermal cycling equipment and thereby significantly decreasing the testing duration.

The principal advantage of this methodology is its integration of enzyme-free molecular self-assembly amplification mechanisms with an immunochromatographic visual readout, thus reducing equipment dependency and improving detection accessibility. In contrast to conventional qPCR, this system is primarily designed to be activated by RNA, which diminishes the need for DNA removal steps and simplifies the preprocessing workflow. Its user-friendly operation and mild reaction conditions enhance its applicability for rapid screening, on-site testing, and application in resource-limited environments.

CHA-LFIA/GICA is presently more appropriate for use as a rapid screening or trend monitoring instrument. However, its stability in complex clinical serum samples, resistance to interference, and compatibility with standardized quantitative systems (including IU/mL reporting) necessitate additional validation. Future advancements in sample preprocessing techniques and improvements in tolerance to matrix interference are expected to enable these immunochromatographic platforms, founded on molecular self-assembly, to serve a complementary function in the rapid screening of pgRNA, monitoring of dynamic therapeutic efficacy, and conducting epidemiological studies.

#### 4.6.2. Isothermal Amplification-Clustered Regularly Interspaced Short Palindromic Repeats (CRISPR)-Coupled Detection System

In conventional RT-qPCR and digital PCR, which rely on thermal cyclers and involve extended detection cycles and encounter difficulties in differentiating RNA isoforms, the advancement of novel pgRNA detection strategies that feature rapid response, isothermal reactions, and structural discrimination capabilities has become an essential focus in contemporary methodological research. Recently, nucleic acid detection systems that combine isothermal amplification technology with CRISPR cleavage reactions have rapidly advanced in virology research, providing novel technical methods for the rapid detection and point-of-care applications of HBV RNA [58].

Zhu et al. [59] developed a novel HBV RNA detection system that combines reverse transcription-multi-enzyme isothermal rapid amplification (RT-MIRA) with CRISPR/Cas13a technology. The RT-MIRA technique enables effective RNA-to-DNA amplification at temperatures ranging from 39 to 42 °C, eliminating the need for thermal cycling equipment and thereby significantly minimizing the overall reaction time [60]. The amplification products generated using RT-MIRA subsequently function as recognition templates for Cas13a. Upon sequence-specific complementary binding between the CRISPR RNA (crRNA) and the target RNA, the collateral cleavage activity of Cas13a is activated. This activation leads to the cleavage of reporter RNA labeled with a fluorophore-quencher, producing detectable signals. By employing primers and crRNA specifically designed for the HBV preC/C region and its splice variants, this system enables the simultaneous detection of total pgRNA and spliced pgRNA within a single-tube reaction, allowing discrimination between different RNA isoforms.

From a methodological standpoint, the RT-MIRA-Cas13a system demonstrates significant advantages due to its isothermal properties and ability to differentiate between isoforms. This system facilitates detection using basic isothermal equipment or portable heating modules through meticulous primer and crRNA design, thereby minimizing dependence on costly instruments. The dual-target strategy provides a novel method for examining HBV RNA composition and monitoring cccDNA transcriptional activity. Furthermore, the low per-sample detection cost, approximately $5 USD, highlights its potential applicability in primary-level laboratories and resource-limited settings [12].

CRISPR-based detection systems are currently more suitable for rapid screening, subtype differentiation, and research-focused dynamic monitoring. This is mainly attributable to their performance being significantly influenced by factors including crRNA design, the efficiency of isothermal amplification, and the conditions of the sample matrix. Although machine learning techniques can predict crRNA mismatch tolerance or enhance crRNA structures to improve specificity and stability, these advancements continue to encounter challenges in interfacing directly with standardized quantitative systems, such as those reported in IU/mL. Collectively, RT-MIRA-Cas13a represents an advanced detection method, offering distinct advantages regarding speed, accessibility, and RNA isoform resolution. It serves as a valuable complement to existing pgRNA quantification systems rather than a replacement.

Overall, rapid and visual detection systems employing isothermal amplification, immunochromatography, and CRISPR technology provide significant benefits regarding reduced equipment dependency, simplified operational procedures, and improved detection efficiency. These characteristics render them especially suitable for application in specific contexts, including resource-limited situations, primary-level laboratories, and point-of-care testing. However, these systems mostly yield qualitative or semi-quantitative results, presenting significant challenges regarding metrological traceability, uniformity of amplification target regions, and coverage of pgRNA structural heterogeneity. These limitations impede their direct integration with standardized quantitative platforms. Thus, ensuring comparability and biological consistency in test results and preserving accessibility to detection has become a critical challenge in the clinical translation of pgRNA detection. Therefore, it is essential to further investigate strategies for achieving comparability and standardization in pgRNA test results, focusing on target region selection and the integration of multiple indices.

### 4.7. Target Region Optimization and Comprehensive Detection Strategies

#### 4.7.1. Metrological Traceability and Target Region Optimization for Serum HBV RNA

As the detection of HBV pgRNA transitions from research settings to clinical applications, simply enhancing detection sensitivity is insufficient to facilitate its widespread adoption as a biomarker. Instead, ensuring the comparability of results across various laboratories and detection platforms has become a pivotal methodological challenge that must be resolved for the clinical translation of pgRNA. Despite advancements in automated detection technologies, the measurement of pgRNA remains significantly influenced by factors such as primer-probe design, RNA structural variability, and the lack of standardized reference materials. These issues constrain the capacity to interpret results consistently across different studies and platforms.

To mitigate this critical bottleneck, Yu et al. [61] established a standardized method for detecting serum HBV RNA utilizing TaqMan real-time quantitative RT-qPCR. This method establishes a metrological foundation for pgRNA quantification by incorporating a traceable quantitative reference system. The study was pioneering in its use of the national reference material GBW(E)091049 (HBV DNA national reference material), provided by the National Institute of Metrology (NIM), to generate HBV RNA standards through in vitro transcription. This innovation facilitated the creation of a traceable standard curve system, thereby ensuring methodological traceability for the quantitative analysis of serum HBV RNA.

The study systematically compared the performance of three amplification target regions–preC/C, SF, and XR-regarding sensitivity, accuracy, and detection consistency. The findings indicated that the preC/C target region surpassed the SF (2.94 × 102 copies/mL) and XR (8.82 × 102 copies/mL) protocols across key metrics, including a lower limit of detection (approximately 1.18 × 102 copies/mL), improved linear correlation, and greater stability at various treatment time points. Notably, the test results from the preC/C target region exhibited an optimal linear correlation with the concentrations of national reference standard materials, suggesting that this region is more reliable in reflecting the transcriptional activity of cccDNA based on current evidence.

This study constitutes a pioneering advancement in the domain of serum HBV RNA detection, simultaneously improving two critical dimensions: “metrological traceability” and “target region system optimization.” It provides a methodological paradigm for multicenter comparative studies and the formulation of commercial detection reagents. Methodologically, this work represents a transition in pgRNA quantification from a merely “detectable” stage to a novel phase characterized by “comparability and traceability.” This advancement lays the foundation for the future establishment of standardized pgRNA detection protocols and a framework for clinical interpretation.

#### 4.7.2. Multiple-Indicator Parallel Detection Strategy

In the management of CHB, NAs effectively inhibit viral replication, frequently reducing serum HBV DNA levels to undetectable levels. However, cccDNA frequently remains within the nuclei of hepatocytes [8]. This residual cccDNA can sustain a low level of transcriptional activity, generating small quantities of pgRNA and incomplete reverse transcription products, which may contribute to viral relapse following the cessation of treatment and ongoing antigen expression. Therefore, relying exclusively on a single HBV DNA indicator is insufficient for comprehensively assessing whether viral replication is genuinely suppressed.

In response to this limitation, a study has presented a highly sensitive multi-assay nucleic acid monitoring platform [62,63]. This platform can simultaneously detect HBV DNA, pgRNA, and total nucleic acids (TNA = DNA + pgRNA) in a single experimental workflow. This facilitates the simultaneous assessment of viral replication status across various nucleic acid levels. The system combines real-time fluorescent quantitative PCR/RT-qPCR with semi-quantitative gel analysis, facilitating the detection of trace viral signals even within low viral load ranges that are typically deemed “negative” by standard commercial assays. The research findings suggest that HBV pgRNA can be identified in the majority of patient samples even when serum HBV DNA is undetectable, indicating that “virological suppression” does not equate a complete cessation of replication activity.

Regarding methodological specifics, Yan et al. performed a comparative analysis of various primer-probe designs and found that assays targeting the HBc region demonstrated the highest sensitivity for pgRNA detection, outperforming those targeting the HBx or poly(A) regions. This observation is consistent with the molecular characteristic of pgRNA, which is susceptible to RNase H degradation at its 3′ end during the reverse transcription process [47]. These findings highlight the dual influence of target region selection on sensitivity and biological interpretation, thereby aligning methodologically with the previously mentioned target region optimization strategy focused on the preC/C region.

Regarding methodological specifics, Yan et al. performed a comparative analysis of several primer-probe designs and determined that assays targeting the HBc region exhibited the highest sensitivity for pgRNA detection, surpassing those targeting the HBx or poly(A) regions. This observation aligns with the molecular characteristics of pgRNA, which is susceptible to RNase H degradation at its 3′ end during the reverse transcription process [47]. These findings highlight the dual impact of target region selection on sensitivity and biological interpretation, thereby methodologically aligning with the previously referenced target region optimization strategy focused on the preC/C region. Through the integration of parallel detection methods for HBV DNA, pgRNA, and TNA, this highly sensitive multiplex detection platform integrates a multidimensional assessment framework for analyzing residual replication states during CHB treatment. This approach indicates a significant advancement in pgRNA detection, transitioning from single-marker methodologies to more comprehensive strategies.

## 5. Challenges and Future Perspectives in pgRNA Detection

With the progression of research, serum HBV pgRNA has emerged as a candidate biomarker for reflecting intrahepatic cccDNA transcriptional activity and for supporting treatment-response assessment; however, its clinical interpretability remains constrained by heterogeneous assays, limited standardization, and the lack of prospective, outcome-linked validation. As research advances, serum HBV pgRNA is widely recognized as a vital molecular biomarker that indicates intrahepatic cccDNA transcriptional activity, evaluates antiviral treatment efficacy, and guides decisions regarding treatment cessation. Notwithstanding its biological potential, the integration of pgRNA from a research instrument into routine clinical testing encounters significant challenges. These challenges mostly involve methodological heterogeneity in detection techniques, inconsistencies in result interpretation, insufficient standardization, and poorly defined clinical application scenarios. The distinct significance of pgRNA in clarifying viral replication mechanisms, advancing immune regulation studies, and assessing therapeutic interventions offers extensive opportunities for future research endeavors.

### 5.1. Challenges in Methodology and Standardization

From a clinical-implementation standpoint, the obstacles in pgRNA measurement are not equally consequential. The most critical barrier is the lack of standardization and metrological traceability, which prevents inter-laboratory comparability and regulatory-grade interpretation. The second tier includes RNA–DNA discrimination and target-region heterogeneity, because they directly affect analytical specificity and between-assay agreement. Pre-analytical variability (collection, transport, storage, freeze–thaw definitions) represents a third, but still important, tier that can be mitigated through harmonized handling protocols. This prioritization implies that further incremental gains in analytical sensitivity are unlikely to translate into clinical value unless comparability and specificity are addressed first. Accordingly, as a conservative recommendation for clinical implementation and multicenter studies, samples should be aliquoted at initial processing to minimize repeated freeze–thaw, the definition of a freeze–thaw cycle (temperature range and thaw duration) should be standardized, and freeze–thaw history should be recorded and reported as minimum metadata.

Currently, there is considerable variability in the reported positivity rates of pgRNA among various studies, with values ranging from 20% to 80%. This variability highlights the sensitivity of test results to methodological conditions [47]. Key factors contributing to this variation include inconsistent detection systems, divergent sample preprocessing techniques, insufficient RNA stability, interference from residual DNA, and the lack of standardized quantitative reference materials. Additionally, pgRNA is especially prone to degradation. Notably, Ohlendorf et al. [31] reported a 1–2 log decline in HBV RNA after repeated freeze–thaw in plasma/serum, highlighting that stability can be assay- and protocol-dependent. Interpreted alongside studies reporting limited loss under automated workflows, these findings suggest that freeze–thaw effects are sensitive to sample matrix, baseline viral load, and the operational definition of a cycle (e.g., thaw duration and time at ambient temperature), rather than representing a uniform intrinsic property of pgRNA. This degradation is affected by the sample matrix, the definition of freeze–thaw cycles, and the detection platforms employed, leading to inconsistent conclusions across studies. Furthermore, the anticoagulant type, transport temperature, and storage duration significantly affect test outcomes. These findings highlight that sample processing and preservation conditions are critical preanalytical variables that affect the reliability of pgRNA quantification.

Residual HBV DNA interference represents a major technical challenge that can result in false-positive outcomes. Nguyen et al. [64] developed a one-tube selective RT-PCR method that simultaneously facilitates DNA removal and reverse transcription amplification within a single reaction system, significantly minimizing DNA residue interference. This method exhibits approximately 40% more detection specificity than standard RT-qPCR methods. These innovations offer clinical laboratories a viable strategy to improve detection specificity and simplify procedural workflows.

Moreover, the lack of standardization in primer design and the selection of amplification target regions further reduces the comparability of research findings. Wu et al. [65] reported that commonly used primers typically target the 5′ core region or the poly(A) region. The pgRNA levels detected across different target regions can vary by 1–2 logarithmic scales, with certain primers potentially co-amplifying preC mRNA, leading to an overestimation of quantitative results. Laras et al. [66] reported that in HBeAg-negative patients, approximately 96.6% of serum RNA is composed of pgRNA, with the remainder being preC mRNA. Without proper distinction, this can interfere with the accurate quantification of pgRNA. Therefore, clearly defining the molecular origin and biological relevance of the target region in the detection design, and utilizing dual-target or differentiated strategies when necessary, are essential prerequisites for enhancing detection accuracy. Accordingly, when poly(A)-proximal targets are used, studies should either justify why preC mRNA contribution is negligible in the intended population or adopt dual-target/differential strategies to constrain co-amplification, and explicitly report target-region coordinates to support cross-study interpretability.

The primary obstacle impeding the clinical translation of pgRNA is the insufficient standardization within the field. Currently, there is no World Health Organization (WHO)-endorsed international reference standard for HBV RNA. Consequently, numerous studies present their findings using disparate units, such as copies/mL, log10 copies/mL, or IU/mL, resulting in cross-laboratory variations of 0.3–0.7 log [43]. Despite previous attempts to standardize pgRNA quantification using HBV DNA standards, the complete eradication of systematic bias remains difficult. The “Hybrid Template Calibration Strategy” introduced by Yan et al. [62] proposes a two-way calibration system that integrates HBV DNA and pgRNA standards, thereby offering a novel method to enhance inter-laboratory consistency in test results. However, further validation is necessary to confirm its widespread applicability.

### 5.2. Technological Innovations and Methodological Breakthroughs

Recently, pgRNA detection technology has been transitioning from experimental exploration to clinical application. The primary focus of development has been on optimizing detection workflows, improving specificity, and advancing multi-index integration. Nguyen et al. [64] developed a one-tube selective RT-PCR technique that concurrently achieves DNA removal and reverse transcription amplification, thereby markedly improving detection specificity and minimizing operational complexity. Concurrently, the multi-target detection platform developed by Yan et al. [62] facilitates the simultaneous quantification of HBV DNA, pgRNA, and TNA within a single workflow. This innovation enhances a comprehensive assessment of viral replication and transcriptional status. These methodological advancements improve detection sensitivity and reproducibility and lay a technical foundation for future applications in standardization.

### 5.3. Clinical Integration and Prospects for Therapeutic Evaluation

At the level of clinical application, there is a growing body of evidence indicating that individual biomarkers are inadequate for accurately representing the underlying transcriptional activity of cccDNA. The comprehensive analysis of pgRNA in conjunction with markers such as HBcrAg and HBsAg is increasingly acknowledged as an essential strategy for evaluating disease status, treatment efficacy, and the risk of treatment discontinuation. The “cccDNA Activity Scoring Model” proposed by Wu et al. [65] has demonstrated superior efficacy in predicting treatment response and the risk of relapse post-discontinuation when compared to single biomarkers. Further research by Wang et al. [67] and Laras et al. [66] confirmed the advantages of multi-index combined analysis in risk stratification and the evaluation of therapeutic efficacy.

Bazinet et al. [68] reported that the “target not detected” status of pgRNA frequently coincides with the simultaneous clearance of HBsAg and HBcrAg. This observation suggests that the transcriptional inactivation of cccDNA may enter a phase of functional silencing. Therefore, the clearance of pgRNA is emerging as an essential observational endpoint in the pursuit of a functional cure. Furthermore, innovative therapeutic strategies, including those based on RNA interference and capsid assembly modulators, consistently employ pgRNA reduction as a primary indicator of efficacy, highlighting its central role in the evaluation of cure-oriented therapies. Future research should prioritize the development of standardized protocols for target region recognition and preanalytical processes, clarifying the functional significance of pgRNA splice variants, and validating multi-marker prediction models. Collectively, pgRNA detection is evolving from a research tool for monitoring viral transcription toward a candidate biomarker that may inform cure-oriented studies and treatment-response assessment; however, its translation into precise clinical decision-making remains conditional on standardized pre-analytics, traceable calibration, and prospective validation across diverse clinical settings. Based on the clinical-translation filter applied in this review, platforms with automation and traceability (or an explicit route to traceability) should be prioritized for multicenter clinical studies, as inter-laboratory comparability and regulatory-grade interpretation remain the key bottlenecks. It represents a crucial connection between molecular virology research and the clinical management of chronic hepatitis B, thereby advancing the steady progress toward a functional cure.

## Figures and Tables

**Table 1:** Summary of various storage and handling conditions for various clinical specimens.

Sample Type	Sample Handling Requirements	Storage Temperature	Storage Time	Reference
**Human plasma or serum**	Snap-frozen in liquid nitrogen and aliquots prepared	−80 °C	Long-term, about 2–3 years	[27]
	Preservative solution such as RNA later or equivalent	23–25 °C	Short-term: approximately one week	[27]
**Lysate or extracted total RNA (includes pgRNA)**	Diluted in a stabilizing agent such as RNA storage solution and divide into equal portions	−80 °C	About 2–3 years	[27]
**HBV pgRNA in plasma**	——	4 °C and 25 °C	2 days	[29]
	4 freeze–thaw cycles, 22 h frozen and 2 h thawed at 25 °C	−20 °C or −80 °C	About 4 days	[29]
**HBV pgRNA in plasma**	——	−20 and 4 °C	30 days	[34]
	——	25 and 37 °C	7 days	[34]
	3 freeze–thaw cycles, −80 °C frozen + 30 min thawed at 25 °C	−80 °C	About 3 days	[34]

Stability outcomes across studies should be interpreted in the context of assay platform, target region, sample matrix, baseline pgRNA concentration, and the operational definition of a freeze–thaw cycle (e.g., thaw duration and time at ambient temperature). Cross-study differences do not necessarily indicate intrinsic biological instability of pgRNA.

**Table 2:** Cross-platform Comparison of Detection Platforms.

Detection Platform/Method	Technical Principle	Limit of Detection (LOD)	DNA Removal Requirement	Primary Application Scenarios	References
Target Region-based One-step	Reverse transcription and PCR performed in a single tube, targeting pgRNA-specific sequences (e.g., poly(A)/preC regions)	Approximately 10^2^ copies/mL	No	Methodology optimization studies; Selective pgRNA amplification	[35,36,37,38]
Encapsidated RNA detection based on nucleocapsid capture	Anti-HBc antibodies capture nucleocapsids, followed by quantification of encapsidated pgRNA after lysis	Depends on the specific system	Yes (DNase I)	Mechanism studies; Nucleocapsid assembly and antiviral drug screening	[39]
Simultaneous Amplification and Testing system (SAT)	Integration of RNA extraction, reverse transcription, and amplification detection into a fully automated closed system	Approximately 50 copies/mL	No	Routine clinical testing and high-throughput screening	[40,41,42]
Fully automated standardized platform with metrological traceability	Fully automated nucleic acid extraction and RT-qPCR, with results calibrated against international standards (IU/mL)	Approximately 1020 IU/mL	No	Reference laboratories; multicenter studies; long-term follow-up	[43]
Highly sensitive automated detection platform	Automated dual-target RT-qPCR improves sensitivity through optimized extraction volume and algorithm integration	Approximately 1022 copies/mL	No	Low viral load monitoring; treatment efficacy and discontinuation assessment	[37]
Multiplex overlapping digital PCR (ddOTs)	Droplet digital PCR with multiple overlapping target regions covering diverse pgRNA transcripts	Approximately 0.4 copies/μL	No	High-resolution absolute quantification; transcript heterogeneity analysis	[25,37,44]
Catalytic hairpin assembly based immunochromatographic test	Catalytic hairpin assembly signal amplification combined with lateral flow or fluorescent immunoassays	Approximately 10^2^ copies/mL	No	Grassroots screening; Point-of-care testing; Resource-limited regions	[45,46,47]
Isothermal amplification-CRISPR coupled detection system	Isothermal reverse transcription amplification (RTMIRA) combined with CRISPR/Cas13a detection	Approximately 10 copies/mL	No	On-site rapid detection; Point-of-care testing (POCT); Subtype differentiation	[48,49,50]
Metrological traceability and systematic optimization strategy for target regions	Traceable reference material-based RT-qPCR, systematically optimize amplification target regions (e.g., preC/C)	Approximately 10^2^ copies/mL	No	Cross-platform comparability studies; Standardization of detection	[51]
Multi-indicator parallel detection strategy	Parallel detection of HBV DNA, pgRNA, and total nucleic acid (TNA) in a single workflow	Platform-dependent	No	Residual replication assessment; antiviral mechanism analysis	[42,52]

## Data Availability

No new data were created or analyzed in this study. Data sharing is not applicable to this article.
